# High-dose thoracic radiation therapy for non-small cell lung cancer: a novel grading scale of radiation-induced lung injury for symptomatic radiation pneumonitis

**DOI:** 10.1186/s13014-021-01857-8

**Published:** 2021-07-15

**Authors:** Weronika Maria Szejniuk, Martin Skovmos Nielsen, Zsuzsanna Takács-Szabó, Jacek Pawlowski, Sahar Sulaiman Al-Saadi, Panagiotis Maidas, Martin Bøgsted, Tine McCulloch, Jens Brøndum Frøkjær, Ursula Gerda Falkmer, Oluf Dimitri Røe

**Affiliations:** 1 Department of Oncology, Aalborg University Hospital, Hobrovej 18-22, 9000 Aalborg, Denmark; 2 Clinical Cancer Research Center, Aalborg University Hospital, Aalborg, Denmark; 3 Department of Clinical Medicine, Faculty of Medicine, Aalborg University, Aalborg, Denmark; 4 Department of Medical Physics, Aalborg University Hospital, Aalborg, Denmark; 5 Department of Radiology, Aalborg University Hospital, Aalborg, Denmark; 6 Division of Radiology, Karolinska University Hospital, Stockholm, Sweden; 7 Department of Haematology, Aalborg University Hospital, Aalborg, Denmark; 8 Department of Clinical and Molecular Medicine, NTNU, Trondheim, Norway; 9 Cancer Clinic, Levanger Hospital, Nord-Trøndelag Health Trust, Levanger, Norway

**Keywords:** Radiation-induced lung injury, Radiological radiation-induced lung injury scale, Radiation therapy toxicity, Radiation pneumonitis, Non-small cell lung cancer radiation therapy, Radiation-related pulmonary toxicity

## Abstract

**Background:**

Symptomatic radiation pneumonitis (RP) may be a serious complication after thoracic radiation therapy (RT) for non-small cell lung cancer (NSCLC). This prospective observational study sought to evaluate the utility of a novel radiation-induced lung injury (RILI) grading scale (RGS) for the prediction of RP.

**Materials and methods:**

Data of 41 patients with NSCLC treated with thoracic RT of 60–66 Gy were analysed. CT scans were scheduled before RT, one month post-RT, and every three months thereafter for one year. Symptomatic RP was defined as Common Terminology Criteria for Adverse Events grade ≥ 2. RGS grading ranged from 0 to 3. The inter-observer variability of the RGS was assessed by four senior radiologists. CT scans performed 28 ± 10 days after RT were used to analyse the predictive value of the RGS. The change in the RGS severity was correlated to dosimetric parameters.

**Results:**

The CT obtained one month post-RT showed RILI in 36 (88%) of patients (RGS grade 0 [5 patients], 1 [25 patients], 2 [6 patients], and 3 [5 patients]). The inter-observer agreement of the RGS grading was high (Kendall’s W coefficient of concordance = 0.80, *p* < 0.01). Patients with RGS grades 2–3 had a significantly higher risk for development of RP (relative risk (RR): 2.4, 95% CI 1.6–3.7, *p* < 0.01) and RP symptoms within 8 weeks after RT (RR: 4.8, 95% CI 1.3–17.6, *p* < 0.01) compared to RGS grades 0–1. The specificity and sensitivity of the RGS grades 2–3 in predicting symptomatic RP was 100% (95% CI 80.5–100%) and 45.4% (95% CI 24.4–67.8%), respectively. Increase in RGS severity correlated to mean lung dose and the percentage of the total lung volume receiving 5 Gy.

**Conclusions:**

The RGS is a simple radiologic tool associated with symptomatic RP. A validation study is warranted.

**Supplementary Information:**

The online version contains supplementary material available at 10.1186/s13014-021-01857-8.

## Introduction

The majority of patients with non-small cell lung cancer (NSCLC) develop radiological signs of radiation induced lung injury (RILI) after high-dose thoracic radiation therapy (RT) [1–3]. The incidence of RILI varies from 13–100% [1–4] depending on the RT technique, observed lower (5–25%) in intensity-modulated RT (IMRT) or volumetric modulated arc therapy (VMAT) compared to three dimensional (3D) conformal RT [5]. The clinical manifestations of RILI range from mild pulmonary symptoms to severe radiation pneumonitis (RP) [1]. Clinical RP of all grades is seen in 8–57% of cases, usually occurring 4–12 weeks after RT, and typically requires steroid treatment for several weeks [1, 2, 6, 7]. Severe RP is observed in 6–8% of cases [2, 8–10]. In the acute phase of RILI, radiological findings include ground-glass opacities and consolidations within or occasionally outside the irradiated areas of lung parenchyma, as well as ipsilateral pleural effusions [1]. In the late phase (six months after RT) atelectasis, lung volume reduction, and fibrosis are common [5, 11, 12].

Currently, there is insufficient information to enable the prediction of RP development and no radiological scales that assess the risk of RP based on grading of RILI are available [1, 2, 6, 7, 13–15]. There are several tools to grade lung toxicity based on clinical and radiological data, such as the Radiation Therapy Oncology Group / European Organization for Research and Treatment of Cancer (RTOG/EORTC) [16], the Common Terminology Criteria for Adverse Events (CTCAE) [17], or the Southwest Oncology Group (SWOG) scoring system [18]. Most grading scales focus on the clinical evaluation of RP [19] with a simple description of the radiological signs of toxicity [3, 16–18, 20–22]. Likewise, there are several explicitly descriptive radiological RILI grading scales that do not include a correlation to clinical outcome [1, 22–25].

The aims of this prospective observational study were to define a novel RILI grading scale (RGS) and to correlate the RGS grades of one-month follow-up CT scans with the clinical development of RP in patients treated with high-dose thoracic RT. Furthermore, the inter-observer variability of the RGS and the association between the pre-treatment dosimetric parameters and the grading of RILI were evaluated.

## Materials and methods

### Patients

**Fig. 1:**
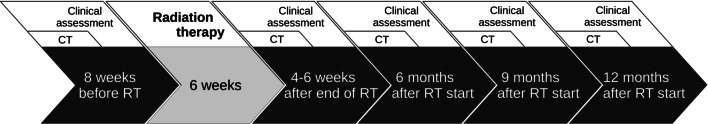
Follow-up schedule (CT, computed tomography; RT, radiation therapy)

This prospective observational study was designed to assess pulmonary features associated with radiation-induced toxicity of thoracic RT. Results describing the association between fractional exhaled nitric oxide and RP in this population have previously been published [7]. Prior treatment with thoracic RT for lung cancer or other malignancies was not allowed. The inclusion criteria for the current study were patients with biopsy-confirmed NSCLC who completed high-dose thoracic RT and were evaluated with at least one CT scan after RT. The follow-up time was defined to 12 months after initiation of RT and was censored at progressive disease (PD) due to the altered schedule of follow-up CT scans. RT toxicity, smoking status, and administration of medications were recorded at the scheduled visits (Fig. 1). Diagnosis of RP was based on CTCAE version 4.0 [17], and grade ≥ 2 was defined as symptomatic RP requiring steroid treatment. The cut-off for early occurrence of RP symptoms was set at 8 weeks (56 days) from the end of RT. Study data were collected and stored in the Research Electronic Data Capture (REDCap) at Aalborg University Hospital. The project was approved by the North Denmark Region Committee on Health Research Ethics (reg. no N-20120029) and reported to the Danish Data Protection Agency (2008–58-0028). Each patient provided written informed consent before the enrolment.

### Radiological imaging

Baseline and follow-up diagnostic CT scans were scheduled within one year (Fig. 1). The CT scans were performed on SIEMENS, GE, or Phillips scanners using a diagnostic thoracic scan protocol (120 kV) with an image slice thickness of 1.25–2 mm. The scans were obtained in portal venous contrast phase were using non-ionic intravenous CT contrast medium, except in patients with hypersensitivity to contrast fluids or decreased renal function. The images were evaluated on both lung window level with width (W) of 1500 Hounsfield Unit (HU) and level (L) of -600 HU (W/L 1500/-600) and mediastinal window level with W/L 420/60 HU. The radiological assessment included a conventional description of RILI and tumour status according to response evaluation criteria in solid tumours (RECIST) version 1.1 [26].

### The novel radiation induced lung injury grading scale (RGS)

The novel RGS was defined based on prior studies describing the most common radiological findings of lung injury induced by RT [1, 23–25]. Typical parenchymal changes and presence of ipsilateral pleural effusion were differentiated into three grades, according to the number of involved lung segments (Table 1). RGS grade 1 (mild) was defined as observation of at least one of the following: ground glass opacities, nodular, patchy, confluent consolidation, volume loss, or pleural thickening, interstitial changes, or fibrosis in < 4 lung segments. Grade RGS 2 (moderate) involved 4–5 segments and grade 3 RGS (severe) involved > 5 segments. Ipsilateral pleural effusion was estimated in the greatest depth transverse to the pleura on a single axial slice. Absence of RILI was defined as RGS grade 0.

**Table 1:** Radiation-induced lung injury grading scale (RGS)

	Number of affected lung segments on thoracic computer tomography scan		
RGS	< 4 segments	4–5 segments	> 5 segments
0	No changes	No changes	No changes
1	Ground glass opacity		
Mild	Consolidation		
	Nodular		
	Patchy		
	Confluent		
	Ipsilateral pleural effusion < 1 cm		
	Volume loss		
	Pleural thickening		
	Interstitial changes		
	Fibrosis		
**2**	Ipsilateral pleural effusion 1–2 cm	Ground glass opacity	
Moderate		Consolidation	
		Nodular	
		Patchy	
		Confluent	
		Volume loss	
		Pleural thickening	
		Interstitial changes	
		Fibrosis	
**3**	Ipsilateral pleural effusion > 2 cm		Ground glass opacity
Severe			Consolidation
			Nodular
			Patchy
			Confluent
			Volume loss
			Pleural thickening
			Interstitial changes
			Fibrosis

### Inter-observer variability of the RGS

After one-year follow-up of all patients, all CT scans were graded according to the novel RGS by a senior radiologist (ZTS). The first follow-up CT scans were used to evaluate the variability of the RGS grades between four senior thoracic oncology radiologists. To evaluate the reproducibility and inter-observer variability of the RGS, no practical training was offered to the radiologists beforehand. Inter-observer communication on RILI evaluation was not allowed and radiologists were blinded to the clinical status of the patients.

### The RGS on the first follow-up CT scan and RP

The RGS grading based on the follow-up CT scans performed 28 ± 10 days after RT, was assessed in relation to incidence and early development of CTCAE grade ≥ 2 RP after RT.

### The RGS and dosimetric parameters

The prescribed mean dose for the clinical target volume (CTV) was 60–66 Gray (Gy) in 30–33 fractions given five times per week. The dose plans were calculated and optimized using the EclipseTM Treatment Planning System (TPS) from Aria® Oncology Information System, Varian Medical System (California, USA). 3D-conformal RT or IMRT were used to deliver the dose. The constraints to the organs at risk and requirement for dose homogeneity followed the Danish national guidelines [26]. The mean lung dose (MLD) and total lung volume receiving 5, 10, 20, 30, 40, 50 and 60 Gy (V5-V60) were extracted from the treatment plans.

### Statistical analysis

Longitudinal data for clinical variables and the RGS grading of all CT scans were collected. Patient characteristics were estimated using descriptive statistics. Fisher’s exact test, the chi-squared test and *t*-test were used to evaluate variances in clinical factors of patients with different RGS grades. Consistency of the RGS scoring between the four independent radiologists was investigated using Kendall’s *W* test. Fisher’s exact test was used to assess the correlation between RGS grades and the occurrence of symptomatic RP (CTCAE grade ≥ 2) as well as between RGS grading and the early onset of symptomatic RP after RT. The change in RGS grading between the first and the second follow-up CT scans was calculated and termed ΔRGS. The association between the dosimetric parameters, occurrence of RILI, and ΔRGS was analysed by one-way analysis of variance (ANOVA). Tests of statistical significance were two-sided and *p *values < 0.05 were considered statistically significant. All statistical analyses were performed using Stata version 14 (StataCorp 2015, College Station, USA).

## Results

### Patient characteristics

**Fig. 2:**
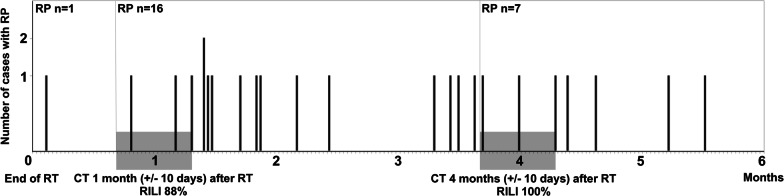
Time-line of radiation-induced lung injury on follow-up computed tomography scans in relation to symptomatic radiation pneumonitis (CT, computed tomography; RILI, radiation-induced lung injury; RT, radiation therapy; RP, radiation pneumonitis)

Between October 2012 and December 2016, 50 patients were included in this study. Nine patients were excluded due to NSCLC-related PD before RT (n = 3), consent withdrawal (n = 4), compliance difficulties (n = 1), and non-RT related death before the first follow-up CT scan (n = 1). A total of 41 patients received thoracic RT and underwent follow-up CT. All patients received a total dose of 60–66 Gy. The majority of patients (n = 32, 78%) were diagnosed with stage III NSCLC (Table 2). Symptomatic RP (CTCAE grade ≥ 2) was observed in 24 patients (grade 2, n = 23; grade 3, n = 1) with an average onset of symptoms 81 days (range, 3–166) after end of RT (Fig. 2). Steroids were used in the treatment of all RP patients, while ten of those patients received both steroids and antibiotics. No patients developed infectious pneumonia requiring treatment with antibiotics only. More than half of the patients (n = 21, 51%) developed tumour progression within one year after thoracic RT.

**Table 2:** Characteristics of the patients

	Characteristics of the patients
	(n = 41)
Age, years	
Median (range)	66 (40–78)
Sex	
Female/male	18 / 23
PS 0	24
PS 1	17
Smoking	
Never	2
Previous	29
Active during RT	10
Histopathology	
Adenocarcinoma	18
Squamous cell carcinoma	14
NSCLC, other type	9
8th TNM stage	
IIA	1
IIB	1
IIIA	11
IIIB	14
IIIC	7
IVA	1
No tumor (post-op. RT)	6
Chemotherapy*	
Concomitant in 3 cycles	31
Induction in 3–4 cycles	8
None	2
Radiation therapy dose	
60 Gy / 30 fx	13
66 Gy / 33 fx	28
Radiation therapy type	
3D conformal	39
IMRT	2
Radiation therapy side	
Left	13
Right	28
Radiation therapy lobe localisation	
Upper	23
Middle/lower	18

PS, performance status; NSCLC, non-small cell lung cancer; post-op. RT, post-operative radiation therapy; TNM, tumour, node, metastasis; fx, fractions; 3D, 3 dimensional; IMRT, intensity-modulated radiation therapy

*Chemotherapy included Carboplatin (i.v.) and Vinorelbine (p.o.)

### Description of RILI changes

**Fig. 3:**
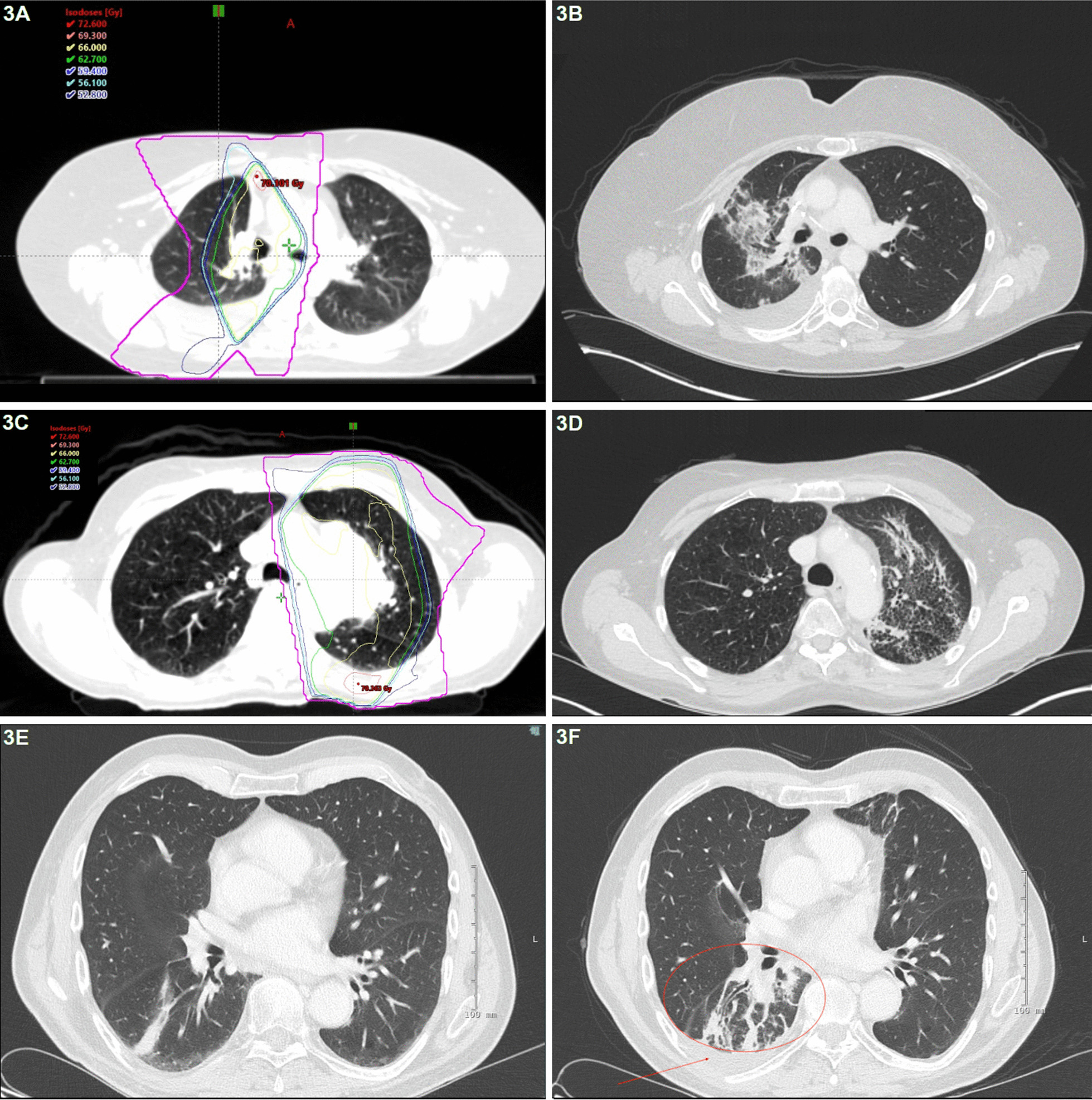
Planning computed tomography (CT) scan with relation to the physical dose distribution. Baseline CT scan before radiation therapy of patient 1 (**a**) and patient 2 (**c**), RGS grade 2 in patient 1 (**b**) and RGS grade 3 in patient 2 (**d**), escalation of RGS in patient 3 on a follow-up CT one month (**e**) and three months (**f**) after RT; *isodose legends: yellow – 66 Gy, green – 62.7 Gy, blue – 59.4 Gy, azure – 56.1 Gy, navy – 52.8, violet – 27 Gy, circle – consolidations, arrow – pleural effusion* (RGS, radiation-induced lung injury grading scale)

All follow-up CT scans were evaluated by a senior radiologist (ZTS) and graded according to the novel RGS. Patients without RILI (RGS grade 0) at the first follow-up CT scan developed RILI on the subsequent CT scan three months later. Thus, all patients developed RILI at some point during the follow-up period (Fig. 2). The most common RILI change was consolidation (n = 27, 66%), but this was not significantly different between patients with and without RP (p = 0.2). Ipsilateral pleural effusion was observed in a minority of patients (n = 7, 17%) and was not significantly different between patients with and without RP (p = 0.1). The occurrence of contralateral RILI changes was more frequently observed in patients with RGS grade 2 and 3 compared to RGS grade 1 (*p* < 0.01) but was not significantly correlated to the development of RP (p = 0.2). Examples of CT scans showing RGS grades 2 and 3 are presented in Fig. 3a–d.

### Inter-observer RGS variability

**Fig. 4:**
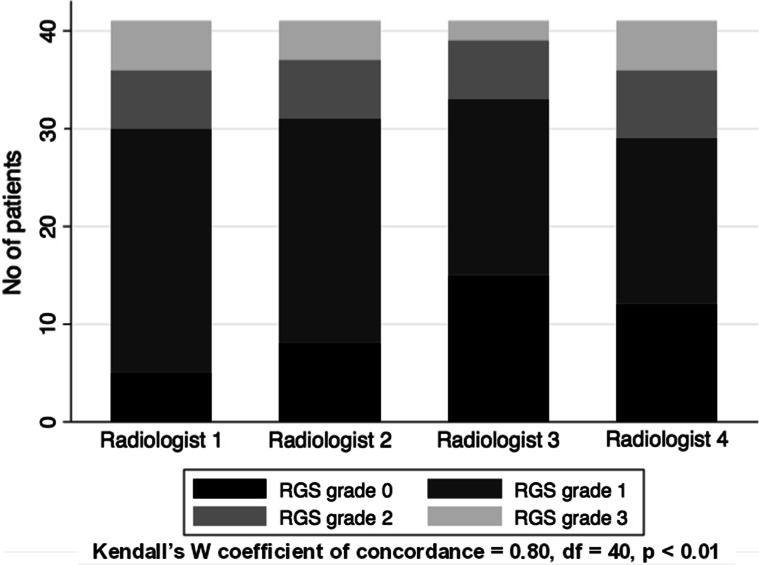
Inter-observer variability of the radiation-induced lung injury grading scale (RGS, radiation-induced lung injury grading scale)

The level of agreement on RGS ranking between the four radiologists was interpreted as good (Kendall’s *W* coefficient of concordance = 0.80, df = 40, *p* < 0.01). The inter-observer variability of the RGS grading showed an agreement of RGS grades 0–1 and grades 2–3 among > 75% of the four radiologists in 90% of cases (Fig. 4).

### Predictive role of RGS at the first follow-up CT scan

**Fig. 5:**
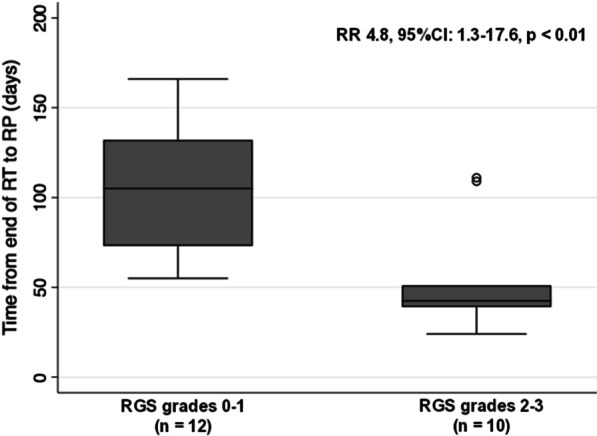
Time from radiation therapy to development of symptomatic radiation pneumonitis in patients with RGS grades 0–1 compared to RGS grades 2–3 based on CT one month after RT (RGS, radiation-induced lung injury grading scale; RT, radiation therapy; RP, radiation pneumonitis; CT, computed tomography; RR, risk ratio)

The first follow-up CT scan was performed on average 29 days (median, 28 days; range 20–50) after RT. RILI changes were observed in 36/41 (88%) patients showing RGS grade 1 (25 patients), 2 (6 patients), and 3 (5 patients), respectively. Two patients were excluded due to RP before the first follow-up CT scan (RGS grade 3, n = 1) and a delayed CT scan 50 days after RT (RGS grade 1, n = 1), respectively. Thus, 39 patients were included in the RP prediction analysis (RGS grades 0–1, n = 29, and RGS grade 2–3, n = 10). RGS grades 2–3 were significantly associated with RP development (relative risk (RR): 2.4, 95% CI 1.6–3.7, *p* < 0.01) (Table 3). The specificity and sensitivity of the RGS grades 2–3 in predicting symptomatic RP were 100% (95% CI 80.5–100%) and 45.4% (95% CI 24.4–67.8%), respectively. The positive predictive value was 100% and the negative predictive value was 58.6% (95% CI 49.2–67.5%), respectively. Patients diagnosed with RP developed symptoms on an average of 81 days after RT (median, 69; range, 24–166). The risk of RP development within 8 weeks after RT was significantly higher in patients with RGS grades 2–3 (median, 42; range, 24–111) compared to patients with RGS grades 0–1 (median, 104; range, 55–166 days) (RR: 4.8, 95% CI 1.3–17.6, *p* < 0.01) (Table 3, Fig. 5).

**Table 3:** Association of symptomatic radiation pneumonitis and RGS assessed on computed tomography scans performed one month after thoracic radiation therapy

Occurrence of symptomatic RP CTCAE grade ≥ 2			Fisher’s exact test
	RGS grade 0–1 (n = 29)	RGS grade 2–3(n = 10)	
RP – (n = 17)	17	0	RR 2.4, 95%CI 1.6–3.7, *p* < 0.01
RP + (n = 22)	12	10	

Onset of symptomatic RP within 8 weeks after RT (n = 22)			
	RGS grade 0–1 (n = 12)	RGS grade 2–3 (n = 10)	
Days, median (range)	104 (55–166)	42 (24–111)	RR 4.8, 95%CI 1.3–17.6, *p* < 0.01
≥ 8 weeks	10	2	
< 8 weeks	2	8	

RGS, radiation-induced lung injury grading scale; RP, radiation pneumonitis; CTCAE, Common Terminology Criteria for Adverse Events; RT, radiation therapy; RR, risk ratio

### Dosimetric parameters

The differences in dosimetric parameters, including MLD and V5-V60, were not statistically significant between patients with different grades of RGS (Additional file 1: Table A.1). However, the comparison between the first and the second follow-up CT scans showed an increase of RGS grade (ΔRGS), representing worsening of RILI changes, in 29 patients (Fig. 3e, f). Seven patients showed no change and one patient showed a decrease in RGS grade. The ΔRGS correlated significantly with dose-volume histogram (DVH) parameters MLD and V5 (Table 4).

**Table 4:** Correlation of ΔRGS based on computed tomography scans performed one and four months after radiation therapy with pre-treatment dosimetric parameters (mean ± SD)

ΔRGS	MLD (Gy)	V5 (%)	V10 (%)	V20 (%)	V30 (%)	V40 (%)	V50 (%)	V60 (%)
1	12.4 ± 3.2	38.4 ± 7.4	30.6 ± 6.5	25.3 ± 6.0	17.2 ± 6.0	21.6 ± 5.4	17.2 ± 6.0	11.7 ± 5.9
2	15.0 ± 2.8	46.3 ± 11.8	35.8 ± 9.9	30.4 ± 7.6	21.3 ± 5.5	26.8 ± 5.8	21.3 ± 5.5	15.4 ± 6.3
3	16.5 ± 2.5	52.8 ± 1.8	41.2 ± 0.2	30.6 ± 5.5	22.5 ± 6.4	26.9 ± 6.3	22.5 ± 6.4	19.1 ± 5.3
0	15.6 ± 3.1	48.7 ± 9.8	36.1 ± 5.6	30.5 ± 4.8	22.7 ± 6.4	27.3 ± 5.2	22.6 ± 6.4	16.6 ± 5.4
*P *value	0.03	0.02	0.08	0.15	0.15	0.05	0.15	0.12

RGS, radiation-induced lung injury grading scale; ΔRGS, change in RGS on computed tomography scans performed one and four months after radiation therapy; MLD, mean lung dose; Vx (%), percent of the total lung volume receiving X Gy

## Discussion

There is an unmet need for a clinically useful tool to predict subsequent RP after high-dose thoracic RT for NSCLC. This study presents a radiological RGS, a novel grading scale for RILI, showing high inter-observer replicability. A high RGS grade, based on follow-up CT scan one month after RT, was significantly associated with a risk of developing symptomatic RP. Hence, the RGS could be a relevant clinical tool to define patients at risk of developing RP, and allowing early RP treatment.

The RGS presented in this study resulted from the need for a simple and reproducible scale to describe the severity of RILI. The main novelty of this RGS was based on the hypothesis that higher numbers of lung segments affected by RILI reflect an increased inflammatory response to RT and thus could determine both the severity of RILI and the occurrence of RP. Previous scales used either general terms to describe RILI (Additional file 2: Table A.2) or the percentage of lung volume affected by RILI [25]. The latter is also empirically based, but is more challenging to quantitate from a radiological perspective. By using lung segments rather than lung volume, the scale is less dependent on the subjective estimation of the radiologists, and provides anatomical accuracy in the description of the extent of RILI. There are several types of RILI changes that may occur either together or separately. We hypothesised that any RILI change was a sign of inflammation and that the severity is more related to the extent, rather than the type of change. Therefore, we did not rank the various RILI changes that can occur, but used the number of affected lung segments. The cut-off at five out of ten anatomical lung segments was empirically based on the radiologists’ experience in ranking RILI severity. The rationale for including an assessment of ipsilateral pleural effusion in the RGS was based on the experience of the radiologists that increased effusion often indicates a more severe inflammatory reaction. The cut-off at 2 cm depth was based on clinical practice of an indication to perform pleural drainage in case of pleural fluid exceeding 2 cm.

There are few published reports of inter-observer variability regarding RILI and RP grading scales [25, 27], but none regarding the prediction of RP. A study by Yamamoto et al. [25] reported a 60% agreement between two observers (Kappa value 0.6). The current study presents the results of an independent evaluation of radiological images by four senior radiologists and shows a relatively high inter-observer agreement (concordance coefficient 0.8). Considering the existence of the high inter-observer variability in radiological evaluation in general [28], the RGS scale showed a satisfactory degree of agreement. Further research is needed to assess the amount of radiological training required to improve the reproducibility of the RGS, with awareness of the lack of a “gold standard” in the description of RILI [29].

The study presents encouraging findings regarding the predictive value of the RGS. Patients in this study developed RP on average 2.5 months after RT, and only one patient developed RP before the first CT scan performed one month after RT (Fig. 2). The RGS grades 2–3 on the first CT scan at one month after RT could predict 10/22 patients developing RP in total and 8/10 patients with RP within eight weeks after RT (Table 3). Importantly, there were no false negatives. Interestingly, most of the cases with RP that were predicted by the RGS grades 2–3, developed the symptoms in the time-window of one month after the first CT scan. This reflects that the timing of the CT scan at four weeks is appropriate for early detection of patients at risk of RP development. Identifying patients showing early signs of RILI, expressed by RGS grades 2–3, could allow distinguishing differential pulmonary conditions resembling RILI, early detection and treatment of RP, and preventing pulmonary fibrosis by hindering development of severe RP. One could speculate if a CT scan at eight weeks would predict the remaining cases of RP. There is no current comparator to this RILI scale and prediction of RP.

The cumulative occurrence of RILI in the study cohort was high, as all patients eventually developed radiological changes either one or four months after RT. This is in line with other studies using similar RT methods [1, 2]. However, according to more recent data of RILI occurrence after modern RT techniques, the incidence of RILI would be lower [5]. The frequency and severity of RILI is multifactorial and related to radiation methods [5, 13, 14], total radiation dose [1, 2], fractionation [6, 15], and dosimetric parameters [1, 15], as well as the RILI scale that is applied [3, 30]. Although the occurrence of RILI seems to be dose-dependent, there are no robust DVH parameters that can predict grade and severity of both RILI and RP [1, 8]. The current study also evaluated changes in RILI on CT scans one and four months after RT. Increasing RGS was associated with MLD and V5 (Table 4). This suggests, that the individual increase in RILI severity depends on the low-dose irradiation triggering late RILI development, detectable several months after RT. In line with this, another study showed that V5 was one of the predictive DVH parameters for the development of radiation-induced lung fibrosis [31].

The incidence of RP ranges between 8–57% in the literature [1, 7, 8, 15]. In the current study, the RP was observed in more than half of patients within 6 months after RT. The relatively high incidence of RP could be explained by the close monitoring of pulmonary toxicity in all patients during the first year post-RT. Furthermore, this cohort was treated with different fractioning and higher total dose compared to other studies of RP after thoracic RT [1, 8, 15], as well as using 3D-conformal RT, resulting in higher incidence of RP compared to IMRT [5, 32]. It can be inferred that the frequent monitoring of the RT toxicity and early RP treatment probably prevented the development of severe RP.

Interestingly, a few patients showed bilateral RILI and most of these developed symptomatic RP. It has been suggested that the existence of immuno-mediated radiological changes in the contralateral lung are associated with the risk of severe RP [9]. Individual radiosensitivity expressed by fulminant RP together with out-of-field RILI was previously observed in a small number of patients after RT [1]. Likewise, it has been proposed that an acute inflammatory response to unilateral lung irradiation with bilateral lymphocytic alveolitis was responsible for the development of hypersensitivity pneumonitis [33]. The unpredictable occurrence of RP unrelated to the irradiated lung volume could be explained by individual hypersensitive immunological response and susceptibility to radiation damage due to inherent factors [7, 34]. In our study of exhaled fractional nitric oxide, patients at-risk for RP had a higher baseline nitric oxide level, indicating a constitutional or genetic susceptibility to RP [7].

The reason for not using one of the existing RILI scales was the lack of quantitative parameters and consensus between them (Additional file 2: Table A.2). Likewise, a definition of RP is difficult due to the use of miscellaneous scales [16–18, 20–22], which may be influenced by other clinical symptoms such as pre-treatment dyspnoea, infectious pneumonia, or chronic obstructive pulmonary disease [35, 36]. The RP diagnosis is based on pulmonary symptoms such as dry cough, dyspnoea or fever after high-dose RT [6]. Radiological imaging can confirm radiation-induced changes but is not required for the diagnosis of RP [1]. Thus, the occurrence of RP can be independent of radiological changes resulting from RT [3]. Furthermore, some of the scales are relatively obsolete, particularly those based on chest X-rays [16, 18]. Since then, the imaging technology has developed substantially. Therefore, there is a need for new studies on RILI and RP in lung cancer using modern radiological and RT technologies. The CTCAE scale was chosen because it only uses clinical assessments of RP and is widely accepted among clinicians.

The strengths of the study were the prospective observational design, frequent radiological and clinical follow-up of RT toxicity, and the independent evaluation of the CT scans by four radiologists. The simplicity and good inter-observer agreement of the RGS makes it appealing for use in clinical trials as a radiological tool describing and grading RILI changes. Furthermore, the RGS could complement the description of RILI severity in trials investigating drugs mitigating RILI and/or RP, as well as in studies performing longitudinal follow-up of RILI resolution. Limitations of the study were the relatively small sample size and the RT method used in the study population. A validation study of RGS feasibility in patients treated with more modern RT techniques is planned in the future as it is expected that incidence of both RILI and RP would decrease after modern RT methods. The low sensitivity and negative predictive value of RGS grades 2–3 can be explained by the fact that clinical RP is also observed in patients with minor radiological changes [1, 2], posing a challenge for the scale. Another challenge in RGS evaluation may be the differential diagnosis between radiation-induced sequelae and the presence of residual tumour. In such cases, it is advisable to compare images with baseline scans. The diagnosis of RP may also be confounded by other pulmonary symptoms, such as infectious pneumonia [35, 36], but this do not seem to have occurred in our study.

## Conclusions

We propose a novel radiological grading scale (RGS) showing a significant association with CTCAE grade ≥ 2 RP in NSCLC patients treated with high-dose radiation therapy. This RGS is a simple tool with high inter-observer agreement. The RGS grades 2–3 observed on CT scans one month after RT defined patients that were at high risk of developing early RP. This provides an opportunity to follow those patients more frequently for timely steroid treatment and describe RILI changes using a systematic structured method. High lung volume irradiated with a low dose was associated with the development of late RILI changes. Further validation of the RGS after IMRT or VMAT in a prospective study is warranted.

## Supplementary Information

**Additional file 1. Table A.1**. Correlation of RGS based on computed tomography scans performed one month after radiation therapy with pre-treatment dosimetric parameters (mean ± SD) (RGS, radiation-induced lung injury grading scale).

**Additional file 2. Table A.2**. Radiation pneumonitis and radiation-induced lung injury grading scales.

## Data Availability

The datasets generated and analysed during the current study are not publicly available due to confidentiality and personal data protection but are available from the corresponding author on reasonable request.
