# Supplementary material for: High-dose thoracic radiation therapy for non-small cell lung cancer: a novel grading scale of radiation-induced lung injury for symptomatic radiation pneumonitis
Source: Radiat Oncol. 2021 Jul 15;16:131. doi: 10.1186/s13014-021-01857-8 (PMC8281688; doi:10.1186/s13014-021-01857-8)
Supplement: Supplementary file 1 — Additional file 1. Table A.1. Correlation of RGS based on computed tomography scans performed one month after radiation therapy with pre-treatment dosimetric parameters (mean ± SD) (RGS, radiation-induced lung injury grading scale). [file 13014_2021_1857_MOESM1_ESM.docx]

Table A.1. Correlation of RGS based on computed tomography scans performed one month after radiation therapy with pre-treatment dosimetric parameters (mean ± SD).

| **RGS** | **MLD (Gy)** | **V5 (%)** | **V10 (%)** | **V20 (%)** | **V40 (%)** | **V50 (%)** | **V60 (%)** |
| --- | --- | --- | --- | --- | --- | --- | --- |
| **0** | 14.5 ± 4.1 | 48.9 ± 7.2 | 38.3 ± 5.5 | 29.2 ± 6.9 | 25.2 ± 8.5 | 20.3 ± 8.4 | 12.9 ± 9.3 |
| **1** | 14.4 ± 3.4 | 44.4 ± 10.9 | 34.6 ± 8.8 | 29.1 ± 7.4 | 25.3 ± 6.3 | 20.1 ± 6.4 | 15.1 ± 5.9 |
| **2** | 13.0 ± 3.3 | 36.6 ± 7.2 | 29.4 ± 6.9 | 25.1 ± 6.0 | 22.9 ± 5.5 | 19.6 ± 5.5 | 13.1 ± 4.8 |
| **3** | 14.4 ± 2.7 | 47.7 ± 9.8 | 34.5 ± 5.1 | 28.8 ± 3.9 | 25.6 ± 4.1 | 20.3 ± 5.7 | 14.1 ± 3.9 |
| **p-value** | 0.8 | 0.6 | 0.5 | 0.5 | 0.6 | 0.8 | 0.3 |

RGS, radiation-induced lung injury grading scale; MLD, mean lung dose; Vx (%), percent of the total lung volume receiving X Gy
