# Supplementary material for: High-dose thoracic radiation therapy for non-small cell lung cancer: a novel grading scale of radiation-induced lung injury for symptomatic radiation pneumonitis
Source: Radiat Oncol. 2021 Jul 15;16:131. doi: 10.1186/s13014-021-01857-8 (PMC8281688; doi:10.1186/s13014-021-01857-8)
Supplement: Supplementary file 2 — Additional file 2. Table A.2. Radiation pneumonitis and radiation-induced lung injury grading scales. [file 13014_2021_1857_MOESM2_ESM.docx]

Table A.2. Radiation pneumonitis and radiation-induced lung injury grading scales.

| Radiation pneumonitis grading scales | | | | | | | |
| --- | --- | --- | --- | --- | --- | --- | --- |
| Scale / grade | Grade 0 | Grade 1 | Grade 2 | Grade 3 | Grade 4 | Grade 5 | Author |
| RTOG / EORTC acute radiation morbidity scoring criteria | No change | Mild symptoms of dry cough or dyspnea on exertion | Persistent cough requiring narcotic, antitussive agents / dyspnea with minimal effort but not at rest | Severe cough unresponsive to narcotic antitussive agent or radiological evidence of acute pneumonitis / intermittent oxygen or steroids may be required | Severe respiratory insufficiency / continuous oxygen or assisted ventilation | --- | Cox et al. 1995 [15] |
| RTOG / EORTC late radiation morbidity scoring criteria | No change | Asymptomatic or mild symptoms (dry cough).  Slight radiographic appearances | Moderate symptomatic fibrosis or pneumonitis (severe cough).  Low grade fever.  Patchy radiographic appearances | Severe symptomatic fibrosis or pneumonitis.  Dense radiographic changes | Severe respiratory insufficiency / continuous oxygen / assisted ventilation | Death directly related to radiation late effects | Cox et al. 1995 [15] |
| EORTC  (LENT-SOMA) | --- | Asymptomatic or mild symptoms; slight imaging changes | Moderate symptoms; patchy imaging changes | Severe symptoms; increased density imaging changes | Severe symptoms requiring continuous oxygen or assisted ventilation | Death | LENT SOMA 1995 [21] |
| NCI-CTCAE version 5.0 | --- | Asymptomatic; clinical or diagnostic observations only; intervention not indicated | Symptomatic; medical intervention indicated; limiting instrumental ADL | Severe symptoms; limiting self-care ADL; oxygen indicated | Life-threatening respiratory compromise; urgent intervention indicated (e.g., tracheotomy or intubation) | Death | Available online: ctep.cancer.gov [16] |
| SWOG | --- | Imaging changes; mild symptoms without steroids | Symptoms requiring steroids or tap for effusion | Symptoms requiring oxygen | Symptoms requiring assisted ventilation | Death | Green et al. 1992 [17] |
| Symptom Only Scale | No increase in lung symptoms | Increase in lung symptoms due to radiation therapy but not requiring steroids | Increase in lung symptoms due to radiation therapy requiring steroids | Increase in lung symptoms due to radiation therapy requiring oxygen | Increase in lung symptoms due to radiation therapy requiring assisted ventilation | Death related to radiation | Aslani et al. 2005 [19]  Faria et al. 2009 [3] |
| Radiation-induced pulmonary symptoms | No symptoms | No intervention taken | Patient treated with medication, typically steroids | Patient requiring oxygen or hospitalization | Death | --- | Marks et al. 2000 [20] |
| Radiation-induced lung injury grading scales | | | | | | | |
| Scale / grade | Grade 0 | Grade 1 | Grade 2 | Grade 3 | Grade 4 | Grade 5 |  |
| Libshitz scale | --- | Homogeneous pattern, slight increase in density uniformly involving irradiated portions of lung | Patchy consolidation within irradiated lung but does not conform to the shape of the radiation field | Discrete consolidation that conforms to the radiation portal but does not uniformly involve it | Solid consolidation totally involves the irradiated lung and contains ectatic bronchi within it | --- | Libshitz et al. 1984 [22] |
| Modified Libshitz scale adapted to 3D-CRT | No change | Slight homogenous increase in radiographic density (e.g. ground-glass appearance) | Patchy or multifocal consolidation that does not conform to the irradiated area | Severe confluent consolidation that conforms to the shape of the irradiated area but does not uniformly involve it | --- | --- | Jenkins et al. 2011 [1] |
| Radiological Grading Scale of Radiation Induced Pneumonitis | No findings | Ground glass opacities without fuzziness of the subjacent pulmonary vessels | The findings may vary from ground glass opacities, extending beyond the radiation field, to consolidations | Clear focal consolidation (+-) elements of fibrosis | Dense consolidation, cicatrisation atelectasis, aerobronchogram and bronchial extension (traction bronchiectasis), significant pulmonary volume loss, and pleural thickening | --- | Kouloulias et al. 2013 [23] |
| CT-based radiological appearance of RILI | None | Slight radiographic appearances | Patchy radiographic appearance | Diffuse radiographic changes < 25% of lung volume | Diffuse radiographic changes > 25% of lung volume | (Death) | Yamamoto el a. 2018 [24] |

SWOG, Southwest Oncology Group; RTOG/EORTC, The Radiation Therapy Oncology Group / European Organization for Research and Treatment of Cancer; LENT-SOMA, Late Effects Normal Tissue Task Force-Subjective, Objective, Management, Analytic; NCI-CTCAE, National Cancer Institute-Common Terminology Criteria for Adverse Events; 3D-CRT, 3-dimensional conformal radiotherapy; CT, computed tomography; RILI, radiation-induced lung injury
